# 64 Epidemiological Features of Carbapenem Resistant Acinetobacter Baumannii Infections in Selected Counties in Tennessee, 2014-2024.

**DOI:** 10.1017/ash.2026.10495

**Published:** 2026-06-23

**Authors:** Lea Widemann, Tiina Peritz, Jane Gould

**Affiliations:** 1 Philadelphia Department of Public Health

## Abstract

**Background:** Antibiotics are among the most frequently prescribed medications in skilled nursing facilities (SNFs), yet an estimated 40-75% of these prescriptions may be inappropriate or unnecessary. Vulnerability of SNF residents to antibiotic resistance, C. difficile, and adverse drug events make antibiotic stewardship programs (ASPs) crucial in these settings. Pharmacists are central to the success of ASPs, yet the scope of their responsibilities within Philadelphia SNFs is not known. Our team designed a survey to evaluate how consultant pharmacists are embedded in SNF ASPs, barriers to providing AS services, and interest in receiving further education. **Methods:** We designed a REDCap survey using the CDC Core Elements for AS in Nursing Homes and the Centers for Medicare & Medicaid Services’ Revised Long-Term Care Surveyor Guidance, which included facility-specific questions for each SNF served, as well as general questions. The survey was disseminated through a multi-modal approach to consultant pharmacists (n=26) identified through previous program activities and all Philadelphia SNFs (n=46) via email, phone, and collaborative meetings. Responses were summarized using descriptive statistics. **Results:** Fourteen pharmacists completed the survey, covering 23/46 (50%) Philadelphia SNFs. Eight pharmacists (57%) reported providing consulting services to <1 SNF in Philadelphia. Six pharmacists (43%) reported having formal training in AS or infectious diseases, and five (36%) served as the lead or co-lead of ASPs, covering 9/23 SNFs (39%). In 83% of SNFs, pharmacists were contracted to provide AS services for <5 hours per week. Limited contracted hours to perform AS activities was the most common consultant-related barrier to providing AS services, reported by 50% of pharmacists. The most commonly reported facility-related barriers were high rate of staff turnover (36%) and staff time constraints (29%). Pharmacists reported supporting a penicillin allergy delabeling program in 17% of SNFs and reported using antibiotic timeouts to assess antibiotic use in 13% of SNFs. Among the 79% of pharmacists interested in receiving further AS education, priority topics were building engagement and collaboration among staff, residents, and leadership (50%), clinical patient care (43%), and tracking, analyzing, and reporting AS metrics (30%). **Conclusion:** Health departments have an opportunity to enhance AS services delivered by SNF consultant pharmacists through educational outreach. Our survey identified penicillin allergy delabeling, initiating antibiotic timeouts, and AS metric reporting as priority topics. Systemic barriers, such as limited time for AS activities and staff turnover, were frequently reported. Addressing these barriers will be essential to enable sustained improvements in SNF ASPs.

